# Photodynamic Diagnosis and Treatment for Atherosclerosis by an Endoscopic Approach

**DOI:** 10.1155/DTE.5.191

**Published:** 1999

**Authors:** Junichi Hayashi, Takashi Saito, Katsuo Aizawa

**Affiliations:** 1 Department of Medicine and Gerontology School of Medicine Kyorin University Shinkawa 6-2072, Mitakashi Tokyo 181 Japan; 2 Department of Biology School of Medicine Kyorin University Shinkawa 6-2072, Mitakashi Tokyo 181 Japan; 3 Department of Physiology Tokyo Medical College Tokyo Japan

## Abstract

The photosensitizer, mono-L-aspartyl chlorin e6 (NPe6), specifically accumulates in the atheromatous plaque. We detected the fluorescence spectra of NPe6 emitted from atheromatous plaques on the descending thoracic aorta by an angioscopic approach using the animal model of atherosclerosis. We also showed that a fluorescence spectrum peak at 675 nm was obtained laparoscopically only in parts of the abdominal aorta with an atheromatous plaque. By a fluorescence endoscope, atheromatous plaques on the carotid artery were recognized as reddish spots from outside the artery. In addition, we visualized specifically at the beating heart surface small coronary atherosclerosis using an epifluorescence stereoscope system.

We examined the effects of photodynamic treatment with NPe6 on the atheromatous plaque. The change in the elastic framework in the atheromatous plaque after photodynamic treatment was evaluated using scanning electron microscopy. The destruction of the architecture of the elastic fiber network in the atheromatous plaque was revealed. We also studied the change in the lipid components of the atheromatous plaque using Fourier transform infrared (FTIR) microspectroscopy. FTIR microspectroscopic analysis showed a dissociation of ester bonds of cholesterol esters in the atheromatous plaque after photodynamic treatment. The framework of the atheromatous plaque and the lipids accumulated in the plaque could be destroyed following such treatment.


*Diagnostic and Therapeutic Endoscopy*, Vol. 5, pp. 191–195
Reprints available directly from the publisher
Photocopying permitted by license only

© 1999 OPA (Overseas Publishers Association) N.V.
Published by license under
the Harwood Academic Publishers imprint,
part of The Gordon and Breach Publishing Group.
Printed in Malaysia.

### Photodynamic Diagnosis and Treatment for Atherosclerosis by an Endoscopic Approach

JUNICHI HAYASHI[a,*], TAKASHI SAITO[b] and KATSUO AIZAWA[c]

[a]*Department of Medicine and Gerontology,* [b]*Department of Biology, School of Medicine, Kyorin University, Shinkawa 6-20-2, Mitakashi, Tokyo 181, Japan;* [c]*Department of Physiology, Tokyo Medical College, Tokyo, Japan*

**The photosensitizer, mono-L-aspartyl chlorin e6 (NPe6), specifically accumulates in the atheromatous plaque. We detected the fluorescence spectra of NPe6 emitted from atheromatous plaques on the descending thoracic aorta by an angioscopic approach using the animal model of atherosclerosis. We also showed that a fluorescence spectrum peak at 675 nm was obtained laparoscopically only in parts of the abdominal aorta with an atheromatous plaque. By a fluorescence endoscope, atheromatous plaques on the carotid artery were recognized as reddish spots from outside the artery. In addition, we visualized specifically at the beating heart surface small coronary atherosclerosis using an epifluorescence stereoscope system.**

**We examined the effects of photodynamic treatment with NPe6 on the atheromatous plaque. The change in the elastic framework in the atheromatous plaque after photodynamic treatment was evaluated using scanning electron microscopy. The destruction of the architecture of the elastic fiber network in the atheromatous plaque was revealed. We also studied the change in the lipid components of the atheromatous plaque using Fourier transform infrared (FTIR) microspectroscopy. FTIR microspectroscopic analysis showed a dissociation of ester bonds of cholesterol esters in the atheromatous plaque after photodynamic treatment. The framework of the atheromatous plaque and the lipids accumulated in the plaque could be destroyed following such treatment.**

*Keywords:* Atherosclerosis, Mono-L-aspartyl chlorin e6, Photodynamic diagnosis, Photodynamic treatment

### INTRODUCTION

Accumulation of several compounds in the porphyrin group such as hematoporphyrin derivative (HpD), dihematoporphyrin ether (DHE, Photofrin II) and hydroxyethyl-vinyldeuteroporphyrin (HVD) in atherosclerotic lesions has been reported [1–4]. Although these porphyrin compounds were considered useful for detecting atheromatous plaques, limitations related to clinical applications such as marked photosensitization of the skin have been pointed out.

* Corresponding author. Tel.: 81-422-47-5511, ext. 3532. Fax: 81-422-44-5310.

Recently, it was shown that mono-L-aspartyl chlorin e6 (NPe6) accumulates specifically and rapidly in atheromatous plaques [5]. Intravenously injected NPe6 disappears with a high clearance rate, making it unlikely to photosensitize the skin. This would make NPe6 a better photochemical material for clinical use than the porphyrin group.

We demonstrated that atherosclerotic lesions could be recognized by analysis of NPe6 fluorescence from within the arterial lumen as well as outside the artery. Then, we showed the effects of photodynamic treatment using NPe6 on atheromatous plaques histologically and biochemically.

### METHODS

Thirty-five New Zealand white rabbits were fed an atherogenic diet containing 0.5% cholesterol for 20 weeks. The photosensitizer, NPe6, was administered intravenously into an ear vein of atherosclerotic rabbits at a dose of 0.5 mg/kg of body weight for photodynamic diagnosis ($n = 20$) and 5 mg/kg for photodynamic treatment ($n = 15$). At 6 h after intravenous injection of NPe6, the vessel was irradiated with a diode laser at a wavelength of 664 nm. The laser power at the catheter tip was controlled to 50 mW for photodynamic diagnosis and to 150 mW for photodynamic treatment.

For photodynamic diagnosis, the fluorescence spectra from the aorta were measured by a fluorescence spectrum analysis system with a dual real time imaging system and a flexible endoscopic catheter [6]. The atheromatous plaque on the carotid artery was checked using a fluorescence endoscope. An epifluorescence stereoscope system was used to visualize atherosclerosis in small coronary arteries [7].

For photodynamic treatment, the energy administered ranged from 50 to 200 J/cm$^2$ of the tissue fluence. The animals were sacrificed by injecting an overdose of pentobarbital 24 h after the photodynamic treatment. The vessel treated was then excised. The effects of photodynamic treatment on the elastic fiber network were evaluated using scanning electron microscopy in parts of the vessel digested by a modified hot alkaline method [8]. Tissue samples were also prepared for Fourier transform infrared (FTIR) microspectroscopic analysis [9].

### RESULTS

The specific fluorescence spectrum peak at 675 nm of NPe6 was detected only in the parts of the abdominal aorta with an atheromatous plaque from outside the adventitia by a laparoscopic approach. It was also noted that the fluorescence spectrum at 675 nm was obtained angioscopically from inside the thoracic aorta only in parts with an atheromatous plaque (Fig. 1).

An atheromatous plaque on the carotid artery was observed as a reddish spot using a fluorescence endoscope (Fig. 2).

It was unable to specify the parts of the coronary arteries which had atherosclerotic changes on the beating heart surface under room light with the naked eye. However, several brightly illuminated branching small coronary arteries were observed clearly against the dark heart surface through the epifluorescence stereoscope (Fig. 3).

Scanning electron microscopic observation revealed damage to the elastic fiber network in atheromatous plaques that was exposed to the laser at 6 h after NPe6 administration, but no significant changes were observed in laser-treated plaques in animals that did not receive the NPe6 pretreatment (Fig. 4).

The infrared spectra measured in the atheromatous plaque using FTIR microspectroscopy exhibited characteristic peaks of cholesterol ester at 1738, 1468, 1380 and 1174 cm$^{-1}$. A marked decrease in the peak intensity at 1738 cm$^{-1}$, which was related to the =C=O ester bond of cholesterol ester, was observed in the treated atheromatous plaques by FTIR microspectroscopic analysis (Fig. 5).

### DISCUSSION

NPe6 accumulated in the atherosclerotic plaque is activated by a laser beam at a wavelength of 664 nm

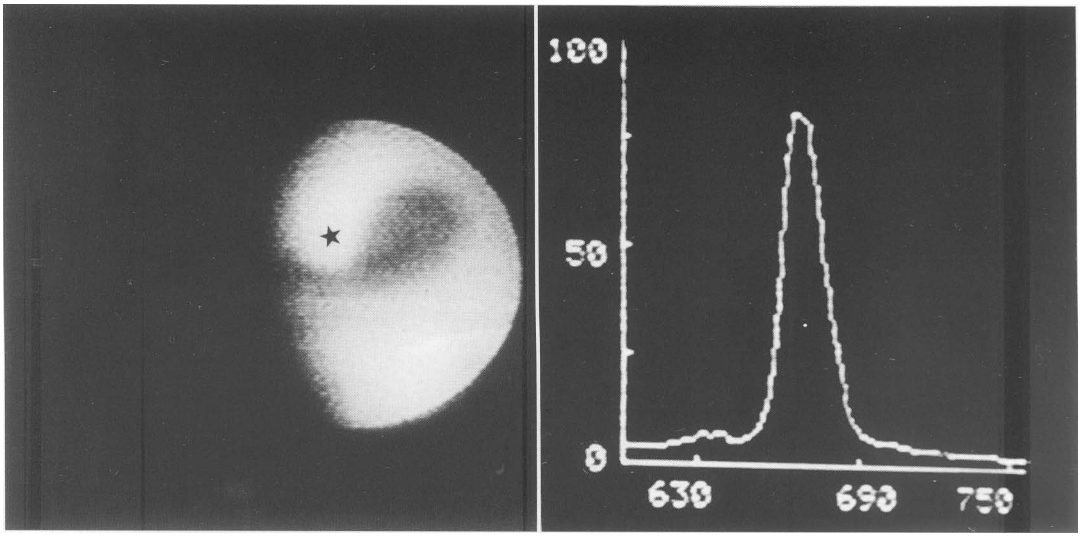

FIGURE 1 The specific spectrum of NPe6 at 675 nm of wavelength obtained from the atheromatous plaque (asterisk) in the aortic cavity by a fluorescence analysis system with the angioscope.

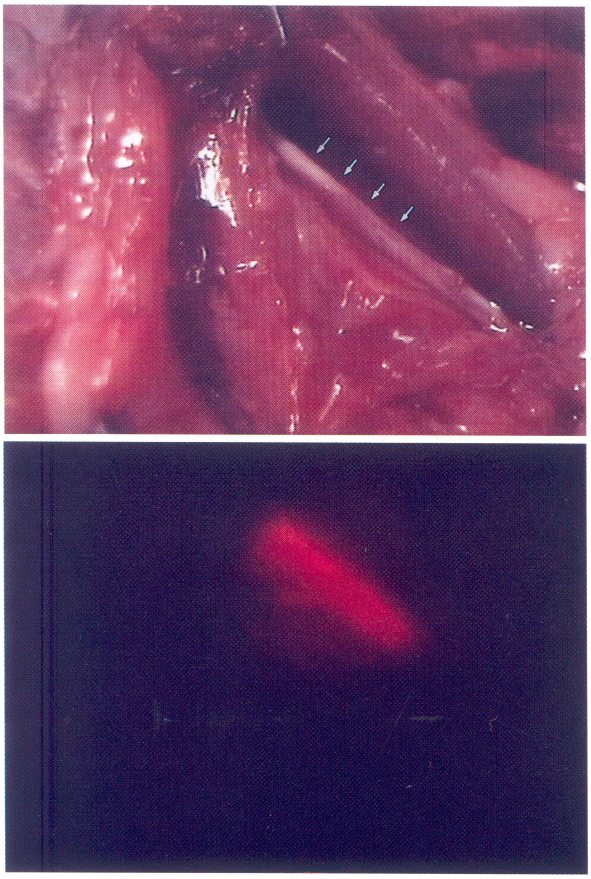

FIGURE 2 The left carotid artery (arrows) was shown under room light with the naked eye (upper panel). The athero-sclerotic lesion was visualized as a reddish spot through the fluorescence endoscope (lower panel).

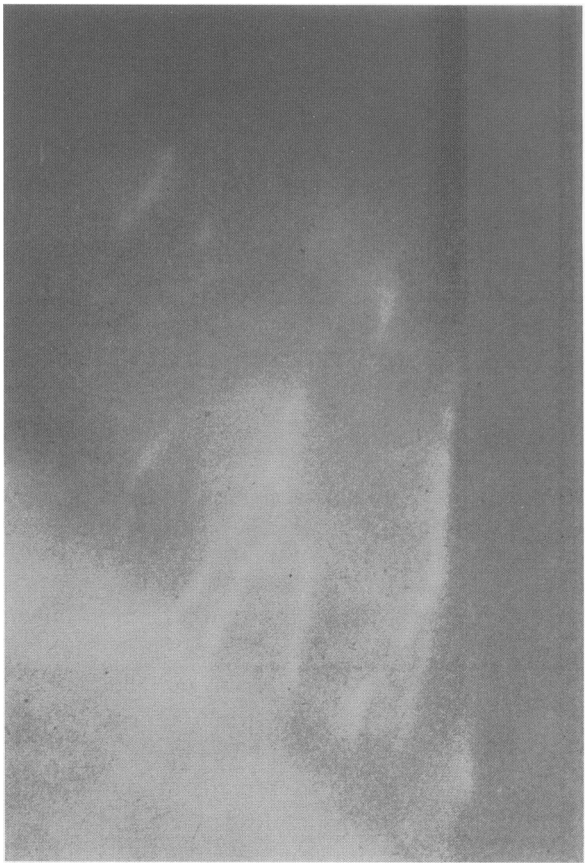

FIGURE 3 Atherosclerosis in small coronary arteries was visualized by an epifluorescence stereoscope system.

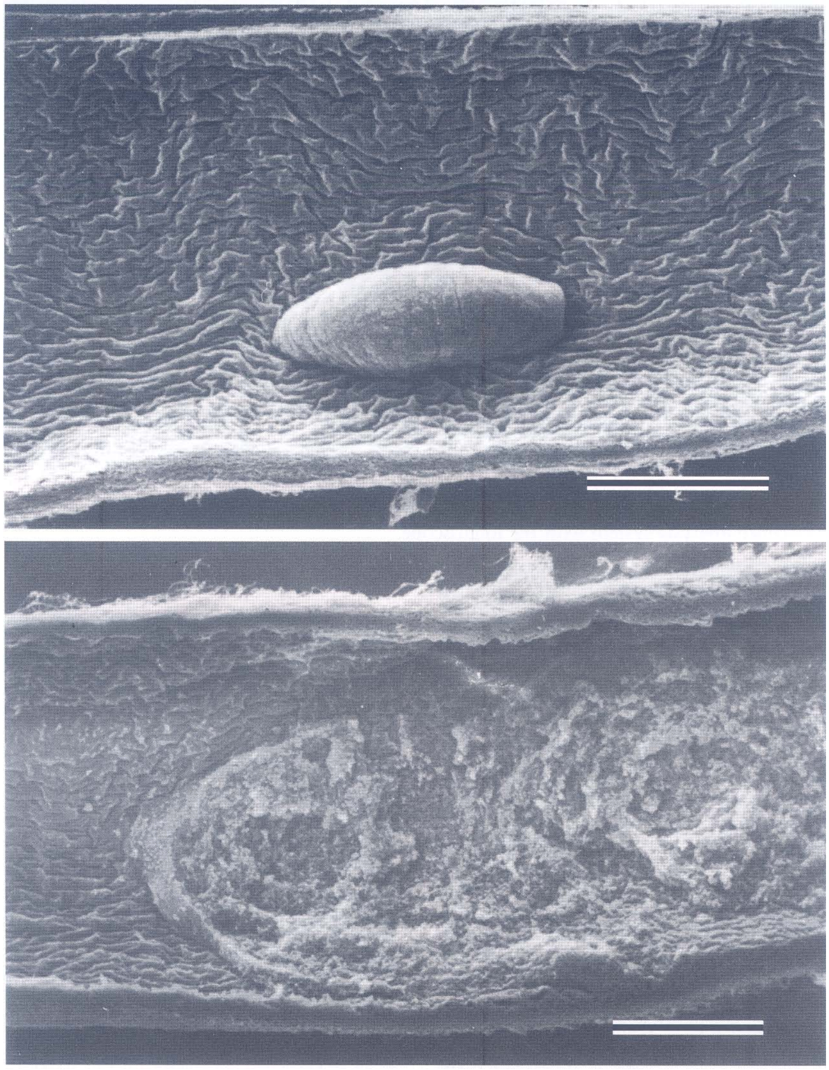

FIGURE 4 Scanning electron micrographs of the lumen of the carotid artery from an untreated rabbit (upper panel) and from a rabbit with photodynamic treatment (lower panel). The untreated atheromatous plaque appears like a hillock on the internal elastic lamina. The architecture of the atheromatous plaque was destroyed after photodynamic treatment. Bar in each panel indicates 500 μm.

that corresponds to the absorption band of NPe6, then NPe6 emits a fluorescence at 675 nm of wavelength. The exciting beam and the emitting fluorescence effectively penetrate the tissue. Thus, we previously reported that NPe6 in the atheromatous plaque was activated from the adventitia of the vessel and detected from outside the vessel [6,7]. In the present study, we demonstrated that the presence and extent of atheromatous plaques was recognized on the several types of the artery. Such information suggests that photodynamic diagnosis with an endoscopic approach may help assess the clinical significance of atherosclerosis.

The principle underlying photodynamic treatment for atherosclerosis is the activation, by laser irradiation, of a photosensitizer that is selectively localized in the atheromatous plaque. The atheromatous plaque, as well as the arterial wall, contains a network of elastic fibers which serves as a main framework to keep morphological structure of

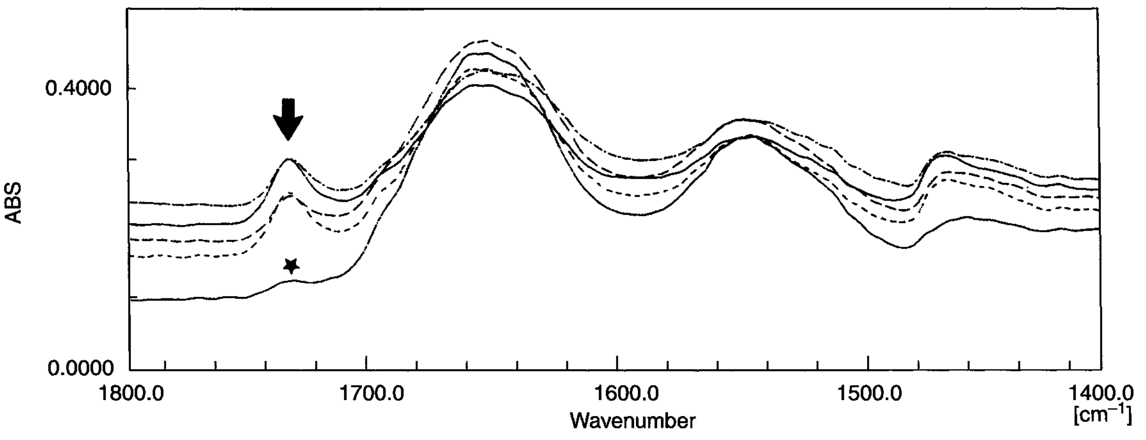

FIGURE 5 Infrared absorption spectra obtained from the non-treated atheromatous plaque showed the specific peak at 1738 cm$^{-1}$ (arrow). After photodynamic therapy with NPe6, the peak intensity at 1738 cm$^{-1}$ was decreased (asterisk).

these tissues. The hot alkaline digestion method is useful in that it removes the non-elastic tissue while preserving the elastic elements, thus allowing one to observe the elastic fiber network following photodynamic treatment [8]. We demonstrated that atheromatous plaques of the carotid artery can be selectively degraded by laser irradiation following pretreatment with the photosensitizer, NPe6. In addition, FTIR microspectroscopic analysis is useful to detect changes in the chemical structure of lipids accumulated in the atheromatous plaque [9]. We showed a dissociation of ester bonds of cholesterol esters, the lipids accumulated in the atheromatous plaque, after photodynamic treatment. Findings suggest that photodynamic treatment with NPe6 on the atheromatous plaque could be an effective clinical approach to regress atherosclerosis.

### References

[1] Spears, J.R., Serur, J. and Shropshire, D. *et al.* Fluorescence of experimental atheromatous plaques with hematoporphyrin derivative. *J. Clin. Invest.* 1983; **71**: 395–399.

[2] Kessel, D. and Sykes, E. Porphyrin accumulation by atheromatous plaques of the aorta. *Photochem. Photobiol.* 1984; **40**: 59–61.

[3] Spokojny, A.M., Serur, J.R. and Skillman, J. *et al.* Uptake of hematoporphyrin derivative by atheromatous plaques: studies in human *in vitro* and rabbit *in vivo*. *J. Am. Coll. Cardiol.* 1986; **8**: 1387–1392.

[4] Delettre, E., Brault, D. and Bruneval, P. *et al. In vitro* uptake of dicarboxylic porphyrins by human atheroma. Kinetic and analytical studies. *Photochem. Photobiol.* 1991; **54**: 239–246.

[5] Yasunaka, Y., Aizawa, K. and Asahara, T. *et al. In vivo* accumulation of photosensitizers in atherosclerotic lesions and blood in atherosclerotic rabbit. *Lasers Life Sci.* 1991; **4**: 53–65.

[6] Hayashi, J., Kuroiwa, Y. and Sato, H. *et al.* Trans-adventitial localisation of atheromatous plaques by fluorescence emission spectrum analysis of mono-L-aspartyl chlorin e6. *Cardiovasc. Res.* 1993; **27**: 1943–1947.

[7] Hayashi, J., Saito, T. and Sato, H. *et al.* Direct visualization of atherosclerosis in small coronary arteries using the epifluorescence stereoscope. *Cardiovasc. Res.* 1995; **30**: 775–780.

[8] Saito, T., Hayashi, J. and Aizawa, K. Acute effects of photodynamic treatment on elastic fiber network in atherosclerotic plaques of rabbit aorta. *Lasers Med. Sci.* 1998; **13**: 126–131.

[9] Hayashi, J., Saito, T. and Aizawa, K. Change in chemical composition of lipids accumulated in the atheromas of rabbits following photodynamic therapy. *Lasers Surg. Med.* 1997; **21**: 287–293.

